# Vitamin D and Indices of Bone and Carbohydrate Metabolism in Postmenopausal Women Subjected to a 12-Week Aerobic Training Program—The Pilot Study

**DOI:** 10.3390/ijerph17031074

**Published:** 2020-02-08

**Authors:** Alicja Nowak, Monika Dalz, Ewa Śliwicka, Helena Elegańczyk-Kot, Jakub Kryściak, Katarzyna Domaszewska, Maria Laurentowska, Piotr Kocur, Barbara Pospieszna

**Affiliations:** 1 Department of Hygiene, Poznan University of Physical Education, 61-871 Poznań, Poland; anowak@awf.poznan.pl (A.N.);; 2 Department of Physiology and Biochemistry, Poznan University of Physical Education, 61-871 Poznań, Poland; sliwicka@awf.poznan.pl (E.Ś.);; 3 Chair of Sport Kinesiology, Poznan University of Physical Education, 61-871 Poznań, Poland; 4 Chair of Motor Organ Rehabilitation, Poznan University of Physical Education, 61-871 Poznań, Poland; 5 Department of Athletics, Strength and Conditioning, Poznan University of Physical Education, 61-871 Poznań, Poland

**Keywords:** vitamin D, carbohydrates metabolism, bone turnover markers, Nordic Walking

## Abstract

The purpose of this study was to assess the effect of Nordic walking training on the indices of bone and carbohydrate metabolism in relation to 25(OH)D levels in postmenopausal women that were subjected to the outdoor systematic physical activity. The study was performed in 10 postmenopausal women, who participated in a 12-week Nordic walking exercise program, taking place during spring months (March to June). Anthropometric and biochemical parameters were measured before and after the training program. Serum concentrations of 25-hydroksycholekalciferol (25(OH)D), parathyroid hormone (PTH), insulin, glucose, osteocalcin (OC), C-terminal telopeptide of type I collagen (CTX), and calcium were determined. After the Nordic walking exercise program, a significant increase in the serum levels of 25(OH)D and CTX and a decrease in body mass, body mass index (BMI), fat mass, and PTH concentrations were observed. The findings of the present study suggest that 25(OH)D, as important metabolic regulator, plays a role in the modification of bone markers’ responses after the outdoor training program, independent of the physical activity effects.

## 1. Introduction

Hormonal changes and lifestyle modifications, which are characteristic for the menopause period, often lead to various impairments of metabolic processes [1,2]. Aerobic exercises are often recommended to elderly or overweight individuals as the prevention of metabolic disturbances and functional limitations [3,4]. Physical activity is also an important factor influencing bone tissue metabolism and it is recommended in the prevention and treatment of postmenopausal osteoporosis. Exercise modality is an important determinant of the bone remodeling effect, although “impact exercises” are usually beneficial for the proper bone tissue function maintenance [5]. Among many different forms of physical activity programs, Nordic walking (NW)—an marching activity with specially designed poles used to push against the ground to activate the upper body while walking—promotes the improvement of body composition, muscle strength, postural control, exercise capacity, and quality of life [6,7,8].

The effect of aerobic physical activity on bone tissue is related to both the action of mechanical loads and modification of systemic factors that are involved in metabolic processes, among others, glucose metabolism and insulin sensitivity [9,10,11]. In the last decade, the interrelation between bone and carbohydrate metabolism has been a particular subject of research [12,13,14]. However, studies assessing the impact of systematic physical activity on bone and metabolic indices should take the significant problem that outdoor physical activity can increase exposure to ultraviolet (UV) radiation into account, which might modify bone and carbohydrate metabolism by altering the serum concentration of 25(OH)D [15], regardless of the influence of the mechanical stimulus.

Vitamin D influences human physiology through calciotropic and non-calciotropic actions. Calciotropic actions are related to bone mass regulation procedures. However, there is also evidence that vitamin D, through vitamin D receptors, plays an important role in many other tissues, including skeletal muscles [16] and adipose tissue [17], and in the regulation of glucose and insulin homeostasis [18]. Moreover, low serum calcidiol (25(OH)D) concentrations, which express the body’s vitamin D status, accompany many types of diseases e.g., cancer, cardiovascular disease, and metabolic and autoimmune disorders [19]. Vitamin D is a novel independent predictor of resting metabolic rate in adults [20] and research that was conducted on animal models has shown that it plays an important role in the regulation of energy balance [21]. It was documented that seasonal variations in serum 25(OH)D concentration, due to changes in UV intensity, lead to changes in energy metabolism [15]. Melin et al. [22] observed that, among elderly people, regular daylight exposure during the summer enhanced 25(OH)D levels and improved calcium homeostasis.

Therefore, the aim of this study was to assess the effect of Nordic walking (NW) training during the spring months (March to June) on indices of bone and carbohydrate metabolism in relation to 25(OH)D levels in postmenopausal women.

## 2. Materials and Methods

### 2.1. Participants

The study included 10 postmenopausal women between 53 to 65 years of age (mean age of menopause 49.4 ± 3.69 years), in whom a Nordic walking (NW) exercise program was applied. Participants with inflammatory disorders, osteoporosis, diabetes mellitus, or who were undergoing hormone replacement therapy were not included in the study. In the research group, three women declared taking multivitamin supplements that contained vitamin D. All of the women provided their written consent to participate in the study. The Local Ethics Committee at the Poznań University of Medical Sciences (no. 216/11 and 830/12) approved the study protocol.

### 2.2. Nordic Walking Training

The Nordic walking exercise program lasted 12 weeks (between March and June), took place outdoors, and was carried out under the supervision of a qualified instructor. Training sessions were repeated three times a week and each lasted 60 min. (5-min., warm-up, 50-min. walking interval, and 5-min. cool-down). The participants were asked to maintain a marching speed, until the intensity of their heart rate, as measured by an exercise tracker (FT1, Polar, Kempele, Finland), corresponded to 90% of their ventilatory threshold (VT), which had been determined earlier. The individual walking distance was measured while using the eTrex 10 gps tracking device (Garmin Ltd., Olathe, KAN, USA) during the first (3400 m) and last training session (4350 m). The average speed of the marching was later calculated to be 4.1 km/h and 5.2 km/h, respectively.

Prior to the program, the ventilatory threshold was estimated in the laboratory while using a cycloergometer (Ergo Metrics 900, Ergoline, Bitz, Germany) [23]. The cardio-respiratory parameters were continuously assessed with the use of a portable breath-by-breath gas analyzer (Oxycon Mobile, Jaeger, Würzburg, Germany) and a heart rate monitor (s610i, Polar, Kempele, Finland). The initial workload (25 W) was increased by 25 W every 3 min., up to VT. The anaerobic threshold for each subject was established based on the change in the ratio of carbon dioxide elimination (VCO_2_) to oxygen consumption (VO_2_), i.e., the respiratory exchange ratio (RER) and the point at which a rapid increase in pulmonary ventilation (VE) occurred, which corresponds to VT.

### 2.3. Anthropometric and Biochemical Measurements

Body mass and height (WPT 60/150.O; Radwag, Radom, Poland) were measured before and after completing the training program. The value of BMI was calculated by dividing body mass (kg) by the square of body height (m^2^). Body composition was assessed in a fasting state while using the bioimpedance method (TANITA BC-418 MA). The fasting blood samples for biochemical tests were taken from the antecubital vein between 7.00 and 10.00 a.m. The serum was separated and stored at −70 °C. Immunoenzymatic enzyme-linked immunosorbent assay (ELISA) methods were used to determine the serum concentrations of osteocalcin (OC, eBioscience, Vienna, Austria; sensitivity, 0.2 ng/mL), C-terminal telopeptide of type I collagen (CTX; Immunodiagnostic Systems, Boldon, Great Britain; sensitivity, 0.02 ng/mL), 25-hydroksycholekalciferol (25(OH)D; Demeditec Diagnostic GmbH, Kiel, Germany; sensitivity 1.9 ng/mL), and parathyroid hormone (PTH; DRG International, Springfield Township, NJ, USA; sensitivity, 1.57 pg/mL). The concentrations of insulin (BioSource Europe, Nivelles, Belgium; sensitivity, 1 μIU/mL) were analyzed while using a radioimmunologic assay. Glucose and calcium (Ca) concentrations were measured with commercially available assays (Cormay, Lublin, Poland), and the homeostasis model assessment of insulin resistance index (HOMA-IR) was calculated [24].

### 2.4. Statistical Methods

All of the statistical analyses were performed while using Dell Statistica (data analysis software system), version 13, downloaded from software.dell.com The normality of the data distribution and homogeneity of variance were verified using the Shapiro–Wilk and Levene’s tests, respectively. The t-test and Wilcoxon test for normally and non-normally distributed variables, respectively, were employed to evaluate the influence of the training program on the assessed indices. The relationship between the variables was tested while using Spearman’s rank correlation. A *p*-value < 0.05 was considered to be significant.

## 3. Results

### 3.1. Comparative Analysis of Somatic Characteristics and Biochemical Indices Measured before and after a 12-Week Aerobic Training Program

Table 1 presents the descriptive statistics of somatic features and biochemical indices that were obtained before and after the completion of the Nordic walking exercise program. Comparative analysis of the data that were obtained before and after the intervention showed a significant decrease in body mass (*p* < 0.05), BMI (*p* ≤ 0.01), fat mass (*p* < 0.05), PTH (*p* < 0.05), and an increase in the levels of 25(OH)D (*p* ≤ 0.01) and CTX (*p* < 0.05). There were no significant differences in the levels of other indicators (Table 1).

### 3.2. Associations between Measured Variables before and after a 12-Week Aerobic Training Program

The analysis of correlations showed significant positive relationships between CTX and OC concentrations before (r = 0.85, *p* = 0.002) and after the intervention (r = 0.73, *p* = 0.0166). Correlations between these markers (resporption and synthesis, respectively) indicate the relationship between both processes of bone metabolism, expressing the bone turnover rate. Before the intervention, the OC level negatively correlated with insulin (r = −0.64, *p* = 0.048) concentration, and OC/CTX ratio with body mass (r = −0.71, *p* = 0.020), height (r = −0.82, *p* = 0.004), and fat mass (r = −0.65, *p* = 0.043). Before and after the intervention, the positive relationships of HOMA-IR with BMI (r = 0.75, *p* = 0.012; r = 0.71, *p* = 0.020, respectively) and fat mass (r = 0.67, *p* = 0.034; r = 0.76, *p* = 0.011, respectively) and after the intervention with body mass (r = 0.69, *p* = 0.026) were found.

### 3.3. Relationships between Variable Changes (Δ_before–after_) during a 12-Week Aerobic Training Program

The difference in CTX concentration (Δ_CTX_), which was caused by the intervention, correlated positively with Δ_25(OH)D_ concentration (r = 0.67, *p* = 0.035) and Δ_HOMA-IR_ (r = 0.79, *p* = 0.007) value. The intervention-induced change in insulin concentration (Δ_insulin_) positively correlated with the insulin level determined before the intervention (r = 0.67, *p* = 0.032).

## 4. Discussion

The surprising finding of the study is that a 12-week NW program caused an increase in the level of bone resorption marker (CTX) in postmenopausal women. We expected the opposite results. Other investigations of exercise programs that were based on marching also delivered diverse effects. Siwapituk and Kitisomprayoonkul [25] concluded that three-months supervised treadmill walking (three times a week) was not sufficient to inhibit the rate of bone turnover in postmenopausal women. In turn, Ma et al. [26] in a meta-analysis suggested that six-months walking therapy exhibit positive effects on femoral neck bone mineral density (BMD) without significant effects on other parts of the skeleton, in peri- and postmenopausal women. In healthy postmenopausal women, which were engaged in 30 weeks of supervised walking at different intensities, Borer et al. [9] found that the walking speed can significantly affect the amount of load exerted on the skeleton, and that bone mass was preserved or slightly increased with speeds above 6.14 km/h. In our training program, the walking speed was much lower than the effective speed that was applied in the mentioned research [9]. However, what is well documented, the use of Nordic walking poles increases the load placed on the skeleton during walking [6,7,8]. Although the Nordic walking with 4.1–5.1 km/h speed, as applied in this study, did not cause favorable changes in the level of bone turnover markers, it cannot be excluded that the use of NW training with higher walking speed could have beneficial effects on bone tissue.

During walking with the additional use of poles, there is a greater skeletal muscle engagement when compared to ordinary walking. Perez-Soriano et al. [27] suggested that NW could be considered an intermediate mode between normal walking and running because of bigger walking stability and higher maximum speeds. Exercises that are characterized by high load dynamics are of particular importance for maintaining high bone mass values and appear to be the most effective in reducing postmenopausal bone loss at the hip and spine [28]. However, in our study, the mechanical stress that was imposed during training was not sufficient for inhibiting the bone resorption rate. We did not notice significant changes within the bone synthesis marker (OC) and to the ratio of OC/CTX (expressing the bone turnover rate) despite the increase in CTX concentrations after the intervention, which shows that the applied NW training did not contribute to significant changes in bone metabolism.

We observed a significant increase in 25(OH)D (*p* ≤ 0.01) and the reduction in PTH (*p* < 0.05) levels in participants’ blood serum after the systematic outdoor activity program, and we suppose that they were associated with seasonal changes in UV radiation [15]. Based on the paradoxical relationship between the increments (∆) in 25(OH)D and CTX concentrations after the intervention (*p* < 0.05), we may presume that, in this study, the metabolic effects that were associated with vitamin D were significant for modifying bone metabolism. Calton et al. [15] found that significant seasonal changes in the 25(OH)D concentrations were associated with altered bioenergetics profile, including resting metabolic rate. Ogata et al. [29], who in patients with type 2 diabetes, showed that the 25(OH)D levels correlated with respiratory quotient (RQ), therefore, the basal metabolic rate also demonstrated the direct correlation between energy expenditure and bone metabolism. Therefore, we cannot exclude that, in the studied women, such significant changes in 25(OH)D concentrations after the intervention were related to changes in CTX levels via the modification of energy profile (we can assume that observing the reduction in fat mass). Other authors previously confirmed the associations of 25(OH)D concentrations with adiposity indices [18,19].

The body mass is a strong determinant of bone metabolism, which might be confirmed by the positive relationship between the OC/CTX ratio and body mass and fat mass values at the beginning of the study. Shapses and Riedt [30], summarizing the results of other authors’ research, suggested that weight loss in postmenopausal women, from 4 to 13% of their body mass, may exacerbate the degradation of bone mass by as much as 1 to 4% in comparison to a group of women maintaining a stable body weight. Nguyen et al. [31] showed that the body mass reduction in lean subjects (<60 kg) could lead to the loss of femoral neck bone mineral density (BMD), whereas no significant bone loss was observed among those who weighed more than 70 kg. Women participating in our research were not obese and we cannot exclude that the weight loss of approximately 2.5% was detrimental to bone metabolism. However, the small size of the intervention group and/or the lack of a control group were limiting factors in this study. The control group of postmenopausal women, analyzed earlier in our laboratory, as a match to women participating in cycloergometer training for eight spring weeks, did not show significant changes in the OC and CTX levels. After an eight-week indoor training program, a significant decrease in the levels of OC, HOMA-IR, and no significant changes in CTX levels were observed in studied women [10].

Osteocalcin is an indicator of bone tissue synthesis, however its high level during skeletal involution reflects intense bone metabolism, leading to increased tissue degradation [32]. In studied postmenopausal women, the positive correlations between OC and CTX concentrations confirm the association between those two bone metabolism processes. The negative correlation between OC levels and insulin concentrations determined before the intervention (*p* < 0.05) presents the association of metabolic status with bone metabolism, and might emphasize the important effect of insulinemia on bone metabolism. In postmenopausal women, the same relationship was noted in our or other authors’ previous studies [10,33]. On the other hand, animal model studies showed that uncarboxylated osteocalcin that was produced by bone cells plays an important role in regulating insulin sensitivity and fat mass [13,14].

Cross-sectional studies provide some evidence that vitamin D influences glucose homeostasis and insulin sensitivity [34]. Calton et al. [15] observed that the seasonal increase in 25(OH)D concentrations from winter to summer led to a significant improvement in insulin sensitivity. In our study, no significant relationships were found between the levels of 25(OH)D and insulin or HOMA-IR. We only observed the tendency to insulin concentrations decrement after the training program and no significant changes in the insulin sensitivity index. However, women participating in this study were not obese and their biochemical markers did not indicate any significant disturbances in carbohydrate metabolism. Previously, it was assumed that the occurrence of insulin resistance is indicated by HOMA-IR values exceeding 2.5 levels [35]. In the participants of our study, the values of HOMA-IR before and after the training program were, on average, 1.8 and 1.7, respectively. Therefore, we did not expect spectacular changes in glucose metabolism after the intervention. The beneficial effects of training concerning carbohydrate metabolism are mainly observed in people with reduced glucose tolerance [36]. The positive relationship between the changes (∆) in insulin concentrations after the NW program with its pre-training levels might confirm this mechanism.

## 5. Conclusions

The most important finding of the study is that vitamin D, as an important regulator of energy homeostasis, might modify the biochemical responses after the outdoor training program independently of the physical activity effects. If the application of low-intensity exercises has no beneficial effects on bone turnover and carbohydrate metabolism in non-obese women, we should consider the individualization of the training program (intensity, volume, and type of exercises), adequate to body content and health needs.

## Figures and Tables

**Table 1:** Somatic characteristics and biochemical indices of women subjected to a 12-week aerobic training program.

Indicator	Before Training	After Training	*p*-Value
Body mass (kg)	65.2 (8.89); 66.3 (62.0–72.0)	63.6 (9.28); 65.3 (59.5–71.0)	0.0026
BMI (kg/m^2^)	25.1 (2.54); 25.5 (24.4–26.5)	24.5 (2.66); 24.5 (23.5–26.1)	0.0030
Fat mass (%)	27.5 (5.24); 28.5 (24.3–30.5)	25.6 (5.59); 26.0 (22.1–29.4)	0.0137
Glucose (mmol/L)	5.0 (1.48); 4.7 (4.2–5.1)	4.9 (0.73); 5.0 (4.3–5.3)	0.6465
Insulin (µIU/mL)	8.9 (2.50); 9.0 (7.2–10.9)	7.9 (2.13); 7.8 (6.7–9.4)	0.0908
HOMA_IR_	1.8 (0.85); 1.8 (1.4–2.4)	1.7 (0.59); 1.8 (1.3–2.2)	0.2578
25(OH)D (ng/mL)	18.6 (13.20); 11.7 (10.5–32.2)	40.4 (9.62); 40.1 (32.0–48.5)	0.0069
PTH (pg/mL)	48.1 (10,62); 51.3 (41.2–54.1)	42.2 (11.37); 43.3 (31.0–50.8)	0.0411
Ca (mmol/L)	1.4 (0.12); 1.4 (1.4–1.5)	1.6 (0.25); 1.6 (1.4–1.8)	0.2429
OC (ng/mL)	8.9 (2.96); 7.9 (7.2–11.7)	8.8 (3.15); 9.0 (7.3–10.7)	0.9255
CTX (ng/mL)	0.6 (0.28); 0.6 (0.4–0.7)	0.7 (0.35); 0.6 (0.5–0.7)	0.0469
OC/CTX	15.9 (3.61); 16.2 (13.0–17.8)	13.8 (3.65); 13.5 (11.8–15.7)	0.2263

Results are expressed as mean (SD); median (interquartile range). *BMI* body mass index, *HOMA_IR_* homeostatic model assessment of insulin resistance index, *25(OH)D* 25-hydroksycholekalciferol, *PTH* parathyroid hormone, *OC* osteocalcin, *CTX* C-terminal telopeptide of type I collagen.
